# An Experimental Performance Assessment of Galileo OSNMA †

**DOI:** 10.3390/s24020404

**Published:** 2024-01-09

**Authors:** Toni Hammarberg, José M. Vallet García, Jarno N. Alanko, M. Zahidul H. Bhuiyan

**Affiliations:** Finnish Geospatial Research Institute, 02150 Espoo, Finland

**Keywords:** Galileo, OSNMA, GNSS, authentication

## Abstract

We present Galileo Open Service Navigation Message Authentication (OSNMA) observed operational information and key performance indicators (KPIs) from the analysis of a ten-day-long dataset collected in static open-sky conditions in southern Finland and using our in-house-developed OSNMA implementation. In particular, we present a timeline with authentication-related events, such as authentication status and type, dropped navigation pages, and failed cyclic redundancy checks. We also report other KPIs, such as the number of simultaneously authenticated satellites over time, time to first authenticated fix, and percentage of authenticated fixes, and we evaluate the accuracy of the authenticated position solution. We also study how satellite visibility affects these figures. Finally, we analyze situations where it was not possible to reach an authenticated fix, and offer our findings on the observed patterns.

## 1. Introduction

During the last decade, major concerns have arisen within the global navigation satellite system (GNSS) community regarding how to improve the robustness and resilience against attacks from counterfeit GNSS-like signals, also known as spoofing. One method to prevent spoofing is by ensuring that the information reaching the receiver is authentic and originating from the legitimate claimed source. Galileo’s OSNMA is designed to enable this at the receiver end in a manner that virtually eliminates the need for a chain of trust with dependence on external third-party services. This service, the first of its kind in the civilian segment, opens the door to many and diverse new applications that require authenticated position.

At present, OSNMA has been in the public observation (PO) test phase for about two years. In this phase, interested users are invited to implement the service at the receiver level, test it, and provide feedback to the European Union Space Program Agency (EUSPA). The Navigation and Positioning Department of the Finnish Geospatial Research Institute (FGI) has created an implementation following the pertinent interface control document(ICD) [1] and the receiver guidelines [2], with the particularity that it is to be executed in a computing platform outside the receiver. This implementation, henceforth denoted as FGI-OSNMA, has been created within the frame of the Horizon2020-funded ESRIUM project, which aims at creating roadwear maps with accurate information about the position and shape of road damage and sending prompt and real-time notifications to drivers and autonomous vehicles with instructions to avoid the damaged areas and route recommendations to even the roadwear [3]. In the ESRIUM project, we rely on Galileo’s services for (a) increasing the positioning accuracy of both the sensor vehicle mapping the road and the end-user vehicle receiving the notifications, and (b) the authentication of the position estimates using Galileo OSNMA in order to increase the security and robustness of the whole solution and to detect possible spoofing attacks.

Despite OSNMA being a relatively new and modern technology still in its test phase, there is already relevant literature related to it encompassing both theoretical work [4,5,6,7,8,9] and practical performance assessments [10,11,12,13,14,15,16,17,18,19,20,21]. In addition to this, there are a few open-source implementations of the OSNMA protocol [22,23,24,25], and some companies already support it in some of their products, such as Septentrio [26].

There is also a vast amount of other literature on GNSS vulnerabilities [27,28,29,30]. Spoofing is but one type of attack against GNSS receivers, not to mention that there are various forms of spoofing. While we do not delve deep into these subjects, it is important to note that the current scope of OSNMA is only in navigation message authentication; hence, it is addressing only certain types of attacks. In different threat models, the user may need to employ various different countermeasures.

This paper expands on the practical OSNMA performance assessments conducted in the previously cited papers, similar to, for example, [10,15], and this paper is an extension of the conference paper [31]. We present operational information and some KPIs of OSNMA, such as a timeline showing relevant authentication events, the number of authenticated satellites (that is, the number of satellites whose navigation message has been successfully authenticated by OSNMA) over time, positioning accuracy when utilizing OSNMA, and the number of satellites transmitting OSNMA data over time. In addition to this, we show the dependency of some of the KPIs on the elevation mask. We also take a closer look at the cases where a satellite fails to reach an authenticated status. More specifically, we examine the possible failure of navigation pages’ cyclic redundancy checks (CRCs) and analyze the cases in which having a low number of satellites transmitting OSNMA data poses problems. Having created an OSNMA implementation, we are in a position to discuss and suggest some practical strategies to optimally handle these cases.

The content of the paper is organized as follows. In Section 2, we provide an overview of the OSNMA protocol, focusing on the details needed to understand the rest of this paper. In Section 3, we then explain the experimental setup. Section 4 presents operational information, KPIs, and other related analysis. We then discuss the results and present our observations in Section 5, and conclude the paper summarizing our findings in Section 6.

## 2. OSNMA Overview

The goal of OSNMA is to enable users to verify that the navigation message received through the signal in space (SIS) is both unmodified and authentic. The OSNMA authentication system is based on the Timed Efficient Stream Loss-tolerant Authentication (TESLA) broadcast authentication protocol [32]. In this section, we provide an overview of the TESLA variant used in the OSNMA protocol. For the sake of clarity, we focus on the main technical details necessary to understand the content of this article. A more comprehensive review of modern TESLA variants can be found in [33], and the full details regarding OSNMA can be found in the official specification documents [1,2].

The TESLA protocol is a method to transmit a sequence of authentication keys through a one-way untrusted communication channel from a transmitter to a receiver. In OSNMA, each key is then used to generate a truncated message authentication code (MAC), called a tag, which authenticates part of the navigation message sent by a satellite in a previous subframe. This key sequence is generated by starting from a random seed $K_{i}$, where *i* is a very large number, and the rest of the keys $K_{i-k}$ are obtained by iterating a cryptographic hash function *h* such that $K_{i-1}=T(h(K_{i}\left|\right|t_{i}\left||\alpha )\right)$, where || denotes the concatenation operation of bit-level representations of the operands, *T* is the truncation operation, $t_{i}$ is the time at which key $K_{i}$ was transmitted, and α is a hash salt that is set in the protocol parameters. Then, the keys $K_{1},K_{2},K_{3},\ldots$ are transmitted one by one at regular time intervals and in reverse order with respect to their generation. Due to this construction, the verification that $K_{i+1}$ is part of the correct key chain is a matter of simple hashing, while, due to the properties of cryptographic hash functions (pre-image resistance and collision resistance), it is practically impossible to compute or forge the next key.

Since the authenticity of the keys is verified using previously authenticated keys, the protocol requires that the receiver has access to a single trusted key $K_{j}$ from the past. Usually, this is the so-called root key. In OSNMA, the root key is transmitted with the SIS along with an Elliptic Curve Digital Signature Algorithm (ECDSA) signature that proves the authenticity of the key. The signature is verified against the Galileo public key, which is available at the European GNSS Service Centre (GSC) website, although this can also be retrieved or renewed via the SIS. The public key is further verified against a Merkle tree, the root of which is meant to be pre-installed in the receiver hardware. Therefore, the OSNMA utilizes a variety of well-tested cryptographic methods yet adapts these to the satellite’s specific use case.

The nominal navigation pages contain 40 bits of OSNMA related data, which are divided into header and root key (HKROOT) (8 bits) and MAC and key (MACK) (32 bits) sections. These data are accumulated over the course of fifteen nominal pages, or one subframe, to form a 120-bit HKROOT and 480-bit MACK messages. The HKROOT contains status updates and the data needed for the initialization, while the tags and keys are contained in the MACK section.

It is important to note that, in practice, not all Galileo satellites will transmit OSNMA data. Instead, the satellites that do transmit OSNMA data will also transmit tags that allow the authentication of navigation messages from other satellites as well. This process is called cross-authentication. The importance of cross-authentication is that it adds redundancy to the system, and, in theory, cross-authentication is not limited to Galileo satellites. In the future, it may be used to authenticate satellites from other constellations as well.

The last thing the reader should understand about OSNMA is that the tags and authentications are associated with so-called authentication date and key delay (ADKD) numbers. The ADKD specifies what part of the navigation message is authenticated by the tag and informs about a potential key delay. ADKD = 0 specifies that the tag authenticates ephemeris, clock, and the status of the satellite. ADKD = 4 specifies that the tag authenticates Galileo constellation (not satellite)-specific timing information. Lastly, ADKD = 12, also known as Slow MAC, authenticates the same data as ADKD = 0 but with an additional 10 subframes delay for the key transmission. For the sake of simplicity, in the coming sections, when we say that a satellite is authenticated, we mean that its ephemeris, clock, and status are authenticated by an ADKD = 0 or ADKD = 12 tag.

Each satellite transmitting OSNMA data transmits tags in a fixed sequence, which spans over two subframes or one minute. This sequence is determined by the so-called MAC Look-up Table (MACLT) value and may change over time, and the possible sequences are described in the OSNMA ICD [1]. During the experiment, the MACLT value was 33, which corresponds to a transmitted tag sequence of 00S, 00E, 04S, 00E, 12S, 00E, 00S, 00E, 00E, 12S, 00E, 12E. Here, the first two characters of the tag identifier specify the ADKD type and the third character specifies whether the tag is for self- or cross-authentication (S = self; E = cross).

## 3. Experimental Setup

The data used in this study were collected with a Septentrio Mosaic X5 receiver loaded with the 4.14.0 firmware (FW) version and connected to a Septentrio PolaNt Choke Ring antenna. The antenna was statically mounted in the roof of a building in Finnish Geospatial Research Institute (FGI)’s premises in Espoo in southern Finland and in an open-sky environment. The groundtruth for the antenna position was calculated by an external positioning service AUSPOS. The data used in the present study were collected between 15 August 2023 and 25 August 2023, with a total duration of approximately ten days. The data were logged at 1 s intervals. This dataset is openly available at [34]. In addition to this, in a few cases, we used a four-day-long dataset from about a year ago, from 24 October 2022 to 28 October 2022 in particular, to draw comparisons and highlight the changes in the OSNMA performance. The experimental setup for this older dataset is the same, with only the time and duration being different. This older dataset was analyzed in more detail in [31].

The X5 receiver makes available the raw 234 bits of a Galileo I/NAV navigation page via the GALRawINAV block, which includes the even and odd pages concatenated after deinterleaving and Viterbi decoding ([26]; Section 4.2.5). The inputs to our OSNMA implementation are these blocks, which are then parsed to obtain the different pieces of information involved in the authentication protocol. Note that the receiver with the referred FW version already supports OSNMA processing, but we used our implementation in this analysis because it provides us more control over the process and better capabilities for in-depth investigation.

All the processing in this paper occurred using our own OSNMA implementation, which we call the FGI-OSNMA [25]. The design and implementation of FGI-OSNMA occurred with special emphasis on its modularity, usability in real time, and integrability as a library in third-party applications. FGI-OSNMA is open-source and is available from the GitHub page of National Land Survey of Finland NLS [35]. The correctness of the implementation has been validated by using the official test vectors published by EUSPA, and by comparing the performance against that obtained with other available OSNMA implementations, such as OSNMAlib [22] or the Septentrio implementation. In particular, the FGI-OSNMA and OSNMAlib provide equivalent authentication results on the EUSPA test vectors.

## 4. Results and Analysis

### 4.1. Authentication KPIs

**Figure 1:**
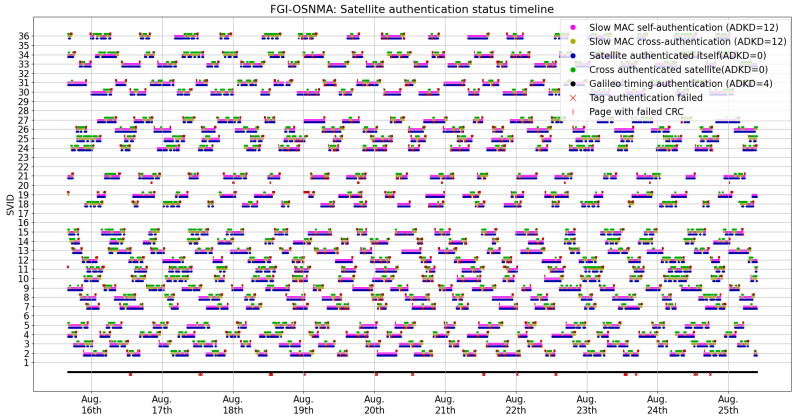
Authentication events over the test period. Note that the red markers slightly below the line are tag authentication failures, while the red markers slightly above the line are CRC failures.

**Figure 2:**
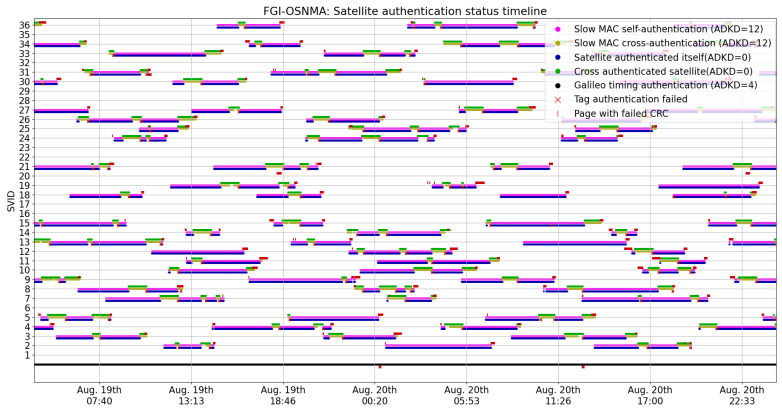
A zoomed version of the authentication timeline.

We now present OSNMA operational information and KPIs pertaining to our tests. Figure 1 shows what we denote as the satellite authentication status timeline. This timeline represents the occurrence of authentication-related events as reported by FGI-OSNMA. In addition to the authentication status and type (i.e., ADKD number), the timeline in Figure 1 shows events where navigation pages were dropped and page CRCs failed. For the sake of clarity, a zoomed version of this figure is presented in Figure 2. We consider that visualizing the occurrence of these events in the graph provides a valuable and informative view of when and how often they can naturally occur. In addition, their occurrence will be analyzed later in this article. We now proceed to present some observed trends and KPIs associated with Figure 1 in more detail.

- The Galileo constellation specific timing information (ADKD = 4) was authenticated 100% of the time. Even though there are a few ADKD = 4 tag authentication failures, because multiple satellites can transmit ADKD = 4 tags, these do not affect the overall authentication status.
- In the authentication scheme, the satellites alternate relatively frequently between self-authentication (which also implies that the satellite is transmitting OSNMA data) and cross-authentication, following a seemingly random pattern. In relation to this pattern, the specification states that it is indeed unpredictable for the user ([1]; Section 5.2).
- There are numerous cases of failed cyclic redundancy checks CRCs. These are associated with poor signal quality. In the dataset used in this study, these occurred exclusively when the satellites were rising over or disappearing below the horizon, in other words, in cases in which satellites have low elevation and therefore poor signal reception quality. It then comes as no surprise that we observed data reception problems from satellites with low elevation.
- Out of the 772,483 events related to authentication (i.e., not the CRCs failures), there are 272 instances where tag authentication failed. In these cases, the received tag consisted only of 0 bits. Given how the tags are generated by a hash function, they should be approximately uniformly distributed, making the reception of such tags in normal conditions close to impossible. This seems to be caused by data reception problems.

**Figure 3:**
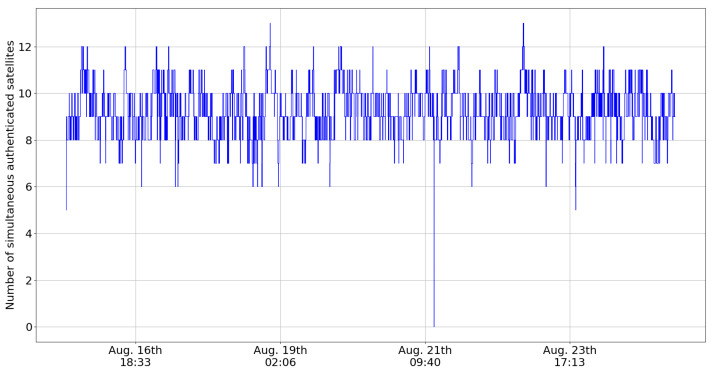
Number of simultaneous authenticated satellites during August 2023.

**Figure 4:**
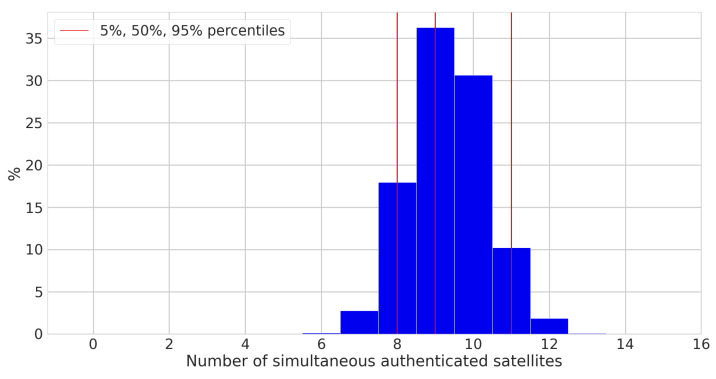
Distribution of the number of simultaneously authenticated satellites available during our tests.

Figure 3 shows the number of simultaneously authenticated satellites over time, and Figure 4 shows the distribution of the number of simultaneously authenticated satellites (that is, the count of satellites with authenticated status at a given time instant), and Table 1 presents some statistics related to these graphs. One important statistic is the percentage of time during which a receiver can compute an authenticated PVT. The condition for this to be possible is that there must be at least four authenticated satellites at the same time. We henceforth use the term authenticated fix to refer to cases where the before-mentioned condition is met. From the statistics presented in Table 1, we observe that there were four or more authenticated satellites visible, and therefore authenticated fixes, 99.996% of the time. Authenticated fixes were available in all but a few outlier cases.

Next, we investigate how the satellite visibility affects the OSNMA performance. We accomplish this by applying an elevation mask. The process is similar to how GNSS receivers discard satellites with low elevation due to high probability of having poor signal quality. We run the OSNMA engine and compute the KPIs using only the received navigation pages from satellites with an elevation higher than the value configured in the mask. This means that both the navigation message and the OSNMA data below the elevation mask value will be filtered out. The effect of the elevation mask in the OSNMA KPIs computed in this manner can be used as an approximation of what could be the expected performance in environments with limited satellite visibility. For example, in urban environments, tall buildings will block the signals coming from satellites with low elevation. The effect of this in the OSNMA performance can be approximated by applying an appropriate elevation mask in the OSNMA processing, as explained before.

**Figure 5:**
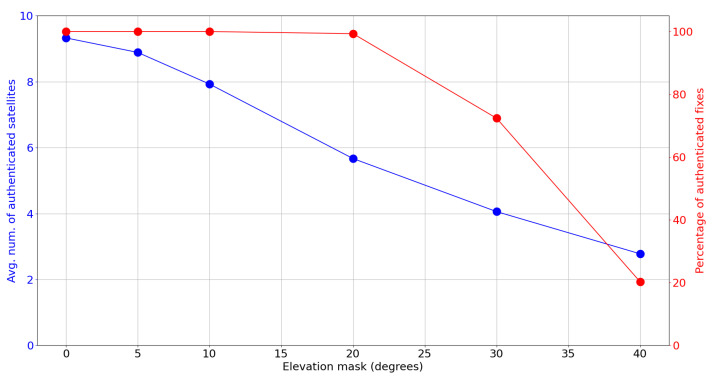
Average number of satellites with authenticated status (blue) and percentage of authenticated fixes (red) as a function of the elevation mask.

Figure 5 shows how the elevation mask affects the average number of authenticated satellites and the percentage of authenticated fixes, and Table 2 presents some related statistics. From the figure, we can observe a gradual and continuous decrease in the percentage of authenticated fixes as the elevation mask increases. The percentage of authenticated fixes decreases slowly at first but rapidly drops as the elevation mask grows.

**Figure 6:**
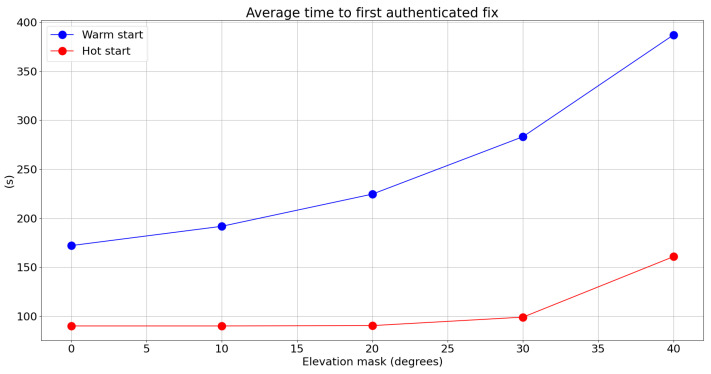
Average TTFAF as a function of the elevation mask.

Figure 6 and Table 3 present the dependency of the time to first authenticated fix TTFAF (that is, how long it would take for a receiver to achieve a first authenticated fix) as a function of the applied elevation mask. The results are computed by running the OSNMA engine over our data one thousand times per elevation mask value, each run starting from a random time point selected from a uniform distribution, and letting the engine run until four satellites become authenticated. Figure 6 graphically shows the average values of these realizations, and Table 3 shows the numerical values of some associated statistics. We present the results for both warm- and hot-start scenarios. In the OSNMA literature, the warm-start scenario refers to the case where the Galileo public key is available to the receiver beforehand. If in addition to this the TESLA root key is available, the scenario is referred to as a hot start. The hot-start case is the most favorable scenario and is also the most likely in practice when the receiver is in frequent use. As we can observe, and similar to other KPIs, the elevation mask can significantly affect the TTFAF. However, the hot-start scenario is visibly less affected until we reach very high levels of elevation mask.

Overall, from Figure 5 and Figure 6, and their respective statistics from Table 2 and Table 3, we see that the OSNMA service and usability can be significantly affected by the satellite visibility. This is of course no surprise as satellite visibility is crucial for any satellite-based application. However, due to its cross-authentication scheme, OSNMA was more vulnerable to poor-visibility conditions [31]. However, during the past year, OSNMA has become more resistant to poor satellite visibility. The reason for this is the increased amount of satellites transmitting OSNMA data, which we will explore in more detail later.

We now proceed to analyze in more detail the cases in which an authenticated fix could not be attained. We note that, in open-sky conditions with no artificial elevation mask applied, authenticated fix was achieved 99.996% of the time. Therefore, these points are more important in limited-satellite-visibility environments.

Some causes of non-authentication are related to the naturally occurring transmission issues: as previously observed, transmission problems can occur during the start or the end of each satellite’s visibility period. However, it is worth noting that this applies to both the satellite transmitting the OSNMA data and the satellite transmitting the navigation data to be authenticated. Corruption in either the navigation message or the cryptographic data will result in failures to authenticate the message. The anomalous case seen in Figure 3, where no satellites were authenticated, was due to corruption in the so-called tag info section of the OSNMA message. This caused the OSNMA receiver to be unable to verify the correctness of the tag sequence. This in turn caused the receiver to discard to tags, as is instructed in the OSNMA receiver guidelines [2].

**Figure 7:**
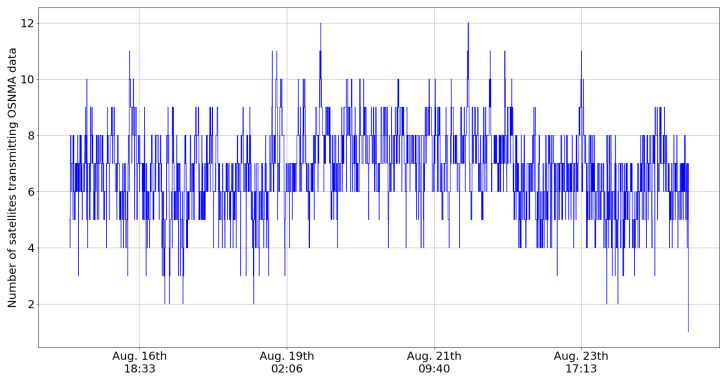
Number of satellites transmitting OSNMA data during August 2023.

**Figure 8:**
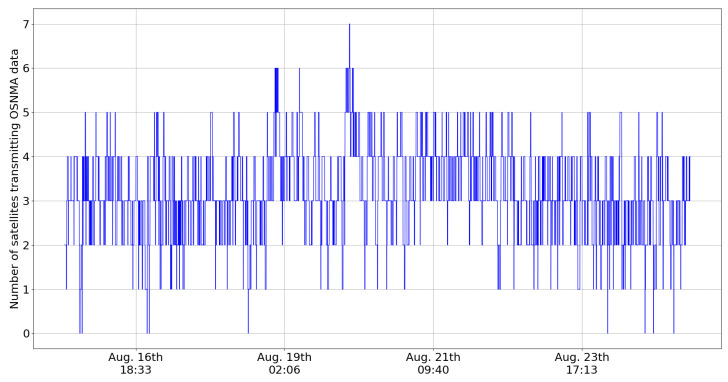
Number of satellites above 30 degree elevation transmitting OSNMA data during August 2023.

The other key aspect impacting authentication is related to the number of satellites transmitting OSNMA data. Figure 7 and Figure 8 present the number of visible satellites transmitting OSNMA data in the no-elevation mask and elevation mask of 30 degree cases. As each visible satellite transmitting OSNMA data will provide at least one authentication (although usually many due to cross-authentication), the situation in Figure 7 enables a high percentage of authenticated fixes. However, the situation is completely different when an elevation mask is applied, as in Figure 8. The number of visible satellites transmitting OSNMA data drops frequently to one or zero, making an authenticated fix impossible. The situation has improved from one year ago, as can be seen from Table 4.

### 4.2. Authenticated Positioning KPIs

In the previous subsection, we studied OSNMA performance from a pure authentication KPI perspective. In this section, we study the effects of using OSNMA in positioning. While we use the term ‘authenticated positioning’, it should be noted that OSNMA at the moment can only authenticate the navigation data, and the PVT is still computed using unauthenticated ranging data. This caveat is also explained in the OSNMA receiver guidelines [2], and there have already been works where the unauthenticated ranging data are exploited to spoof receivers despite authentic navigation data [8]. While the positions we compute in this section cannot be considered fully authenticated, they are resilient against spoofing based on altering the navigation message. Therefore, for simplicity, we still use the term ‘authenticated positioning’, but the reader should be aware of this caveat.

Our procedure to compute authenticated positions is the following. First of all, to make full use of the authentication, we use only Galileo satellites to compute the PVT. In particular, we use only the E1 signal, which is where the OSNMA data are located. We perform the OSNMA processing to obtain the authentication information, and then we filter out the unauthenticated navigation message and the corresponding observables from RINEX navigation and observable files. Therefore, only OSNMA-verified information will be present in the resulting RINEX files. Finally, we use RTKLIB [36] to process these filtered RINEX files to obtain the PVT solution.

The PVT processing configuration we use is very simple: we use the broadcast ionospheric corrections, and the Saastamoinen model for the tropospheric corrections. Just as in the previous section, we apply different elevation masks to simulate different satellite visibility conditions. However, this time, there are two different layers of elevation masks: one for the OSNMA processing, and one for the PVT computation. The PVT mask filters both navigation messages and ranging information from satellites below the elevation mask value. In the tests, we use the same values for these; however, they impact the computation differently. As already discussed, the elevation mask applied to OSNMA processing can reduce the number of visible satellites transmitting OSNMA data, and thus the amount of authentications that are possible. Therefore, the elevation mask in OSNMA processing can have even larger impact than the one applied just in the PVT computation.

**Figure 9:**
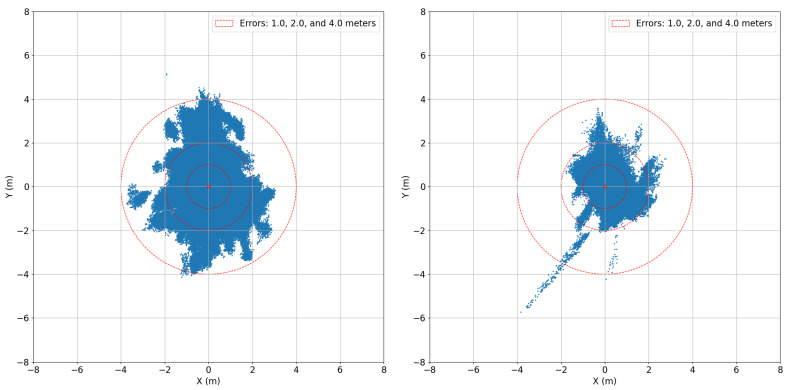
Unauthenticated positioning (**left**). Authenticated positioning (**right**) with no OSNMA processing elevation mask. Both use a 10 degree elevation mask in positioning. The availabilities of the positioning solutions can be found in Table 5.

**Figure 10:**
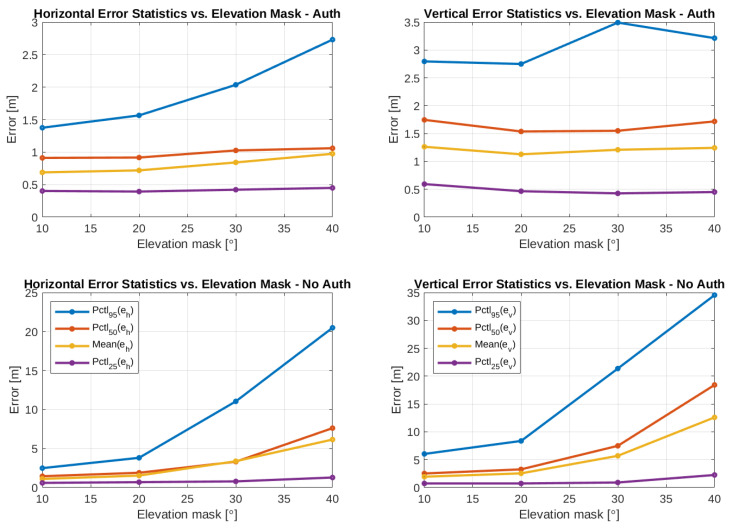
Positioning error mean and percentiles versus the elevation mask.

**Figure 11:**
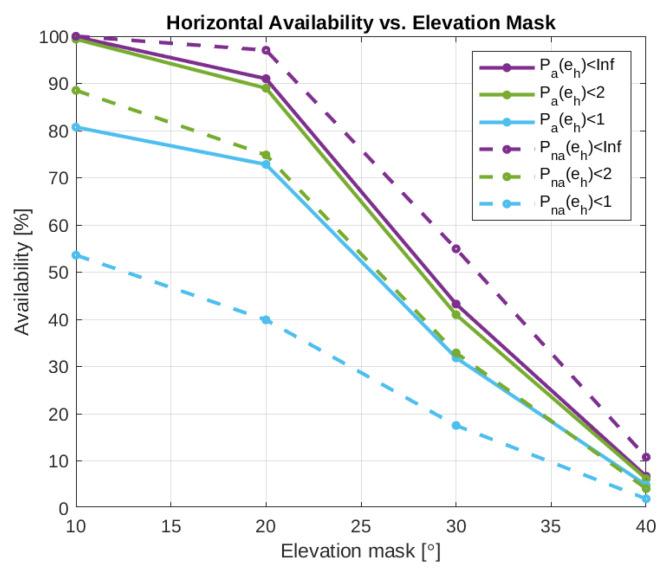
Horizontal availability versus the elevation mask. The colors indicate different error thresholds for the availability. The solid line corresponds to the authenticated positioning solution availability and the dashed line corresponds to the regular non-authenticated positioning solution availability.

Two example ground plots of the test cases are presented in Figure 9 and the results of the processing can be seen in Table 5 and Figure 10. Table 5 presents the 95% percentiles of the horizontal, vertical, and 3D errors together with the 3D RMSE, the average number of satellites used in the PVT computation, and the availability of valid solutions with horizontal errors below one, two, and four meters. The availability of errors less than infinity is added as an indicator of the percentage of epochs during which RTKLIB could compute a solution independently of its error. Figure 10 graphically presents the mean and 25%, 50%, and 95% error percentiles, with the intention for the reader to appreciate how the underlying corresponding distributions change with the elevation mask. In particular, from Figure 10, it can be seen that not only the mean of the positioning error increases as elevation mask increases but also the standard deviation of the error increases. The availability of these position solutions with different error thresholds is visualized in Figure 11. From Figure 11, it can be seen that the overall availability is naturally better in the non-authenticated case. However, with the error threshold of 1 m or 2 m, the availability is better for the authenticated positioning. Unsurprisingly, the results follow a similar trend as already observed in the pure authentication-related elevation mask tests of Figure 5. More interestingly, the authenticated PVT solutions have better accuracy and availability for 1 m compared to the non-authenticated PVT, while suffering only slightly lower overall availability. Interestingly, the epochs where the non-authenticated PVT is available but the authenticated PVT is not correspond to the cases where the positioning error is the highest. This explains the previous observation.

## 5. Discussion

As can be seen from the results, OSNMA enabled authenticated positioning 99.996% of the time in our experiments in open-sky and high-satellite-visibility conditions. With respect to the cases in which it was not possible to reach an authenticated fix, we observed that there were mainly two causes.

First of all, when the satellite elevation is low, the signal quality is degraded, which will cause some navigation pages to be corrupted. Consequently, this will cause some subframes to be incomplete. This is of course not related to the OSNMA specification and similar effects can be expected in any satellite-based application. We highlight that, for real-world applications, it is beneficial that the OSNMA implementation extracts any usable data from the subframe, incomplete or not. Even incomplete subframes are likely to contain useful data. Therefore, it is better to process the data on a page level instead of a subframe level.

We now list a few ways in which dropped pages can affect the OSNMA performance.

- The data in the HKROOT message do not require fast reaction, not to mention that the root key (contained in the HKROOT) message transmission uses redundancy: all the satellites transmitting OSNMA data will transmit the same message, but they transmit the blocks in a different order. This makes the root key transmission both fast and robust. Therefore, the impact of receiving an incomplete HKROOT message from one satellite is not very significant. Some information from the HKROOT message is required to start the authentication process. Therefore, a delay in parsing the HKROOT due to an incomplete subframe will cause a delay in the first set of authentications. However, in the so-called hot-start case (which is the usual one), the receiver has stored a previous HKROOT, and, as long as the TESLA key chain does not change, the receiver can start the authentication immediately without the need to wait for the HKROOT messages. Therefore, moderate navigation page drops have little effect on the HKROOT processing.
- If the key (contained in the MACK message) in the subframe is incomplete, it is not possible to authenticate the previous set of tags immediately. However, all the satellites transmit the same key, not to mention that the receiver may wait for the next key, from which it can recover the missing key with hash iteration. Therefore, page drops affecting the key have minimal effect.
- The tags are the critical part of the transmission: they are the most important part of the authentication process and cannot be recovered later. The tags are naturally independent of each other, meaning that, even if some of the tags are missing due to dropped pages, the others can still be extracted. Also, multiple satellites may transmit a tag for the same satellite. Therefore, OSNMA offers some redundancy for protecting the data. We consider missing tags due to dropped pages to be the worst-case scenario. However, in our experiments, we found barely any problem with this.

The second reason for the failures found during the analysis of our dataset was about the number of visible satellites transmitting OSNMA. This behavior was also noted in [10]. One year ago, this could act as a bottleneck for OSNMA performance, but now the situation is improved, and the effect of this is only noticeable in poor-satellite-visibility conditions.

Another important note is that the application of an elevation mask results in valuable authentication information being discarded from some satellites. In that sense, we note that, while receivers commonly apply a 5–15 degree elevation mask in the tracking and/or PVT computation phases, the same mask should not be applied to OSNMA processing. While the positioning accuracy is known to improve after applying an appropriate satellite elevation mask, for OSNMA, having more data available for processing is better. A low-elevation satellite might still cross-authenticate other satellites.

## 6. Conclusions

In the analysis of our 10-day-long open-sky dataset, we observed that 99.996% of the time a receiver would be able to produce authenticated fixes. This percentage of authenticated fixes naturally decreases in poor-satellite-visibility conditions. Related to this, we observed that, while the cross-authentication scheme of OSNMA has strengths, it can make OSNMA-based positioning somewhat more vulnerable to poor-satellite-visibility conditions. This is because, in poor-satellite-visibility conditions, the OSNMA-data-transmitting satellites might not be visible. This can result in situations where an authenticated fix is not possible even though a regular-position fix is available. Other performance KPIs, such as TTFAF, are similarly affected by satellite visibility. On the receiver side, we highlight that it is beneficial not to discard OSNMA data from satellites with low elevation. While using these satellites in the PVT, computation might not be beneficial; using the OSNMA data that they may carry increases the chances of cross-authenticating visible satellites, which in turn will make more authenticated satellites available to the PVT engine. Also, there are no drawbacks to using OSNMA data from low-elevation satellites: accidentally authenticating a corrupted navigation message is probabilistically close to impossible. 

## Figures and Tables

**Table 1:** Statistics related to the authentication.

Statistic	Value
Simultaneous authenticated satellites: 5% percentile	8
Simultaneous authenticated satellites: average	9.33
Simultaneous authenticated satellites: 95% percentile	11
Percentage of authenticated fixes	99.996%
Self-authentications out of all ADKD = 0 authentications	49.2%
Cross-authentications out of all ADKD = 0 authentications	50.8%

**Table 2:** Percentage of authenticated fixes and percentiles of the number of simultaneous authenticated satellites as a function of the elevation mask.

Elevation Mask	Number of Authenticated Fixes	Authenticated Sats. Count Percentiles: 5%, 50%, 95%
0°	99.996%	8, 9, 11
5°	99.993%	7, 9, 11
10°	99.993%	6, 8, 10
20°	99.319%	4, 6, 8
30°	72.385%	3, 4, 6
40°	20.280%	1, 3, 4

**Table 3:** Percentiles of the TTFAF as a function of the elevation mask in warm- and hot-start scenarios.

Elevation Mask	Warm-Start Percentiles: 10%, 50%, 90%	Hot-Start Percentiles: 10%, 50%, 90%
0°	120, 150, 240	90, 90, 90
10°	120, 180, 270	90, 90, 90
20°	150, 210, 330	90, 90, 90
30°	150, 240, 420	90, 90, 120
40°	180, 300, 690	90, 90, 270

**Table 4:** Statistics related to OSNMA data transmission.

Number of Satellites Transmitting OSNMA Data	August 2023	October 2022
Average	6.78	5.49
0.1% percentile	3	1
1% percentile	4	2
5% percentile	5	3
95% percentile	9	7

**Table 5:** Positioning performance metrics as a function of the elevation mask.

	Elev. Mask [°]	${\mathrm{Pctl}}_{95}(e)$ [m]			RMSE [m] 3D	Av. # Sats.	Horiz. Avail. (P(*e_h_*) < *d*) [%]			
		Horiz.	Vert.	3D			d=1	d=2	d=4	d=∞
Auth	10	1.375	2.795	2.929	1.745	7.062	80.720	99.371	99.870	99.890
	20	1.565	2.749	3.037	1.721	5.220	72.829	88.992	90.837	91.003
	30	2.036	3.493	4.073	2.035	4.321	31.842	40.987	42.902	43.245
	40	2.732	3.213	4.348	2.229	4.087	4.859	6.224	6.572	6.748
No Auth	10	2.490	6.036	6.351	2.937	7.490	53.610	88.524	99.974	100.000
	20	3.814	8.367	9.085	4.341	5.434	39.890	74.832	92.760	97.034
	30	11.049	21.364	24.564	12.094	4.430	17.510	32.898	43.659	54.949
	40	20.489	34.554	45.499	21.522	4.140	1.972	4.122	6.160	10.795

## Data Availability

This data can be found here: https://zenodo.org/records/10259098.
